# Biogenic Silver Nanoparticles as a Strategy in the Fight Against Multi-Resistant *Salmonella enterica* Isolated From Dairy Calves

**DOI:** 10.3389/fbioe.2021.644014

**Published:** 2021-04-26

**Authors:** María Belén Estevez, María Laura Casaux, Martín Fraga, Ricardo Faccio, Silvana Alborés

**Affiliations:** ^1^Área de Microbiología, Departamento de Biociencias, Facultad de Química, Universidad de la República, Montevideo, Uruguay; ^2^Posgrado en Química, Facultad de Química, Universidad de la República, Montevideo, Uruguay; ^3^Plataforma de Investigación en Salud Animal, Estación Experimental INIA La Estanzuela, Instituto Nacional de Investigación Agropecuaria, Colonia, Uruguay; ^4^Departamento de Experimentación y Teoría de la Estructura de la Materia y sus Aplicaciones (DETEMA), Facultad de Química, Centro NanoMat and Grupo Física, Universidad de la República, Montevideo, Uruguay

**Keywords:** antimicrobial, biogenic nanoparticles, *Salmonella* Typhimurium, multi-resistance, Confocal Raman Microscopy

## Abstract

Infectious diseases are one of the most important health problems worldwide, one of the main causes being the development of multi-resistant microorganisms. Likewise, the zoonotic potential of some pathogens and their ability to transfer resistance mechanisms, reduce the therapeutic options in both humans and animals. *Salmonella enterica* is an important pathogen that affects a wide range of animal species and humans, being *Salmonella* Typhimurium one of the most frequent serotypes affecting cattle, causing enteritis, diarrhea, and septicemia. The search for alternative therapeutic approaches has gained importance since the emergence of multidrug resistance to antibiotics and periodic outbreaks of salmonellosis. In this sense, the discovery of new drugs and the development of new strategies, such as the use of nanoparticles with antimicrobial activity, are very promising. The aim of this work was the extracellular production of biogenic silver nanoparticles using fungal extracts and the evaluation of their antimicrobial activity against resistant and multi-resistant *Salmonella* Typhimurium strains. We here demonstrated the potential of the biogenic nanoparticles as effective bacteriostatic and bactericidal agents for use in biomedical applications. In addition, Confocal Raman Microscopy and Atomic Force Microscopy were used to advance the understanding of the antimicrobial mechanism of biogenic nanoparticles against these pathogenic strains, the results of which suggested that the nanoparticles produced damage in several bacterial cell structures.

## Introduction

*Salmonella enterica* isa causal agent of foodborne diseases with a significant impact on public health, affecting both humans and a wide range of animal species. Foodborne diseases cause 33 million deaths a year, affecting 10% of the world’s population. In particular, the pathogenic bacterium *Salmonella enterica* serovar Typhimurium represents a significant threat worldwide, both for food safety and for public health, due to the emergence of multidrug-resistant strains (Wang et al., 2019).

Although more than 2,500 serotypes of *Salmonella* spp. have been reported, only a few affect livestock. In this species, *Salmonella* Typhimurium is one of the most frequent serotypes and etiological agent of enteritis, diarrhea and septicemia (Casaux et al., 2019). In calves, salmonellosis is treated with antibiotics, recommending ß-lactams and sulfonamides in cases of septicemia. Quinolones are used as an alternative treatment when resistance to these antibiotics is confirmed or suspected (Wang et al., 2019). However, the emergence of resistance to quinolones has also been documented (Pribul et al., 2017). In addition, the indiscriminate use of antibiotics, even as food additives for animals, has promoted the appearance and selection of resistant and multi-resistant microorganisms, affecting the efficacy of antibiotic therapy in both humans and animals (Tang et al., 2017). Multi-drug resistance is an emerging problem throughout the world. The misuse of antibiotics is one of the factors responsible for multi-drug resistance in several pathogenic bacteria, such as *Salmonella* Typhimurium (Wang et al., 2019). In South America, this serotype has been isolated from foodborne cases and resistance to beta-lactams has been reported (Cordeiro et al., 2013). In Uruguay, it was shown that *Salmonella* Typhimurium was the most frequent serotype isolated from samples obtained from calves in commercial dairy farms (Casaux et al., 2019). This report included fecal samples from diarrheal calves and organ samples from calves that had succumbed to salmonellosis. This serotype presented the highest resistance patterns and has also been reported in humans, highlighting its zoonotic potential (Casaux et al., 2019).

The appearance of multi-resistance to antibiotics and the periodic outbreaks of salmonellosis are promoting an intense search for new alternative therapeutic developments against pathogenic bacteria. In particular, an important part of this effort is focused on discovering new antimicrobial drugs and new potential alternative strategies to control animal and human diseases (Schäberle and Hack, 2014), such as the use of nanotechnology.

It has been reported that the development of antimicrobials in nanoparticle systems allows to increase the therapeutic effect and overcome side effects, therefore they are considered excellent alternative delivery systems for the treatment of microbial diseases (Youssef et al., 2019). Thus, in recent years the application of nanoparticles for the microbial control has expanded considerably in the area of human and animal health. Different nanoparticles have been effective for the treatment of infectious diseases against antibiotic-resistant bacteria, both in vitro studies and in animal models (Hajipour et al., 2012; Shanthi et al., 2016; George et al., 2019; Thorat et al., 2021). In the area of infection control of veterinary medicine, nanotechnology has a promising role in the prevention or treatment of infections (Youssef et al., 2019).

The mechanisms of antimicrobial action of nanoparticles depend on several factors, including the type of microorganism and the physicochemical characteristics of the nanoparticles (Hajipour et al., 2012). The small size is an important property that influences the nanoparticles uptake and their antimicrobial effect. In particular, the potential application of silver nanoparticles in the treatment of microbial diseases has been reported (Otari et al., 2016). Some silver nanoparticles synthesized by fungi, such as those previously synthesized by our group, presented broad antimicrobial activity against bacteria (Gram negative and Gram positive) and fungi (Rodrigues et al., 2013; Sanguiñedo et al., 2018).

The objective of this work was the extracellular production of biogenic silver nanoparticles using fungal extracts and the evaluation of the antimicrobial activity against resistant and multi-resistant strains of *Salmonella* Typhimurium. In addition, the morphological changes produced in bacterial cells after treatment with nanoparticles and in the phenotypic profile (changes in cellular components) were studied through Atomic Force Microscopy and Raman Confocal Microscopy, as an approach to advance in the knowledge of the antimicrobial mechanism of the biogenic nanoparticles against multi-resistant bacteria.

## Materials and Methods

### Biological Material

For the synthesis of nanoparticles (NPs) the strain of the fungus *Phanerochaete chrysosporium* CCMG 12G from the Cátedra de Microbiología General Collection CCMG, Facultad de Química, Montevideo, Uruguay was used. Previous studies carried out by the group showed the production of stable and high-yield NPs using this strain (Sanguiñedo et al., 2018).

The *Salmonella* Tiphymurium strains used in the evaluation of antimicrobial activity were previously isolated from calves with diarrhea, septicemia and mortality in Uruguay and characterized for their antibiotic-resistance profile (Table 1; Casaux et al., 2019).

**TABLE 1:** Antibiotic-resistance profile of *Salmonella* Typhimurium strains.

**Strain**	**Resistance**	**Intermediate susceptibility**
16-025M4	Cefotaxime, streptomycin, tetracycline	–
16-045M5	Ciprofloxacin, enrofloxacin, streptomycin, tetracycline	–
16-058M4	Ampicillin, amoxicillin- clavulanic acid, trimethoprim-sulfamethoxazole streptomycin, tetracycline	–
16-078M4	Amoxicillin- clavulanic acid, streptomycin, tetracycline	Ampicillin
17-032M21A	Enrofloxacin, gentamicin, streptomycin, tetracycline	Ciprofloxacin
17081M3A	Ampicillin, nalidixic acid, ciprofloxacin, streptomycin, tetracycline	Clavulanic acid
17-197M1	Nalidixic acid, streptomycin, tetracycline	Gentamicin, nitrofurantoin, ciprofloxacin
17-210M7B	Cefotaxime, streptomycin, gentamicin, tetracycline, azithromycin	Trimethoprim-sulfamethoxazole, ciprofloxacin
19-027M2A	Ampicillin, trimethoprim-sulfamethoxazole, clavulanic acid, streptomycin, tetracycline, azithromycin	Amoxicillin- clavulanic acid, ciprofloxacin
16-073M11	Tetracycline	–

### Biological Synthesis of NPs

Cultures were performed by inoculating two plugs of 0.9 cm in diameter of mycelium (grown in Potato Dextrose Agar medium, BD Difco) in flasks with 100 mL of Potato Dextrose Broth liquid medium (BD Difco). Fermentation was carried out at 28°C with agitation on an orbital shaker operating at 150 rpm for 72 h. The fungal mycelium from cultures was filtered and then washed extensively with sterilized distilled water to remove any remaining media components.

The NPs biosynthesis assay was performed as previously described (Sanguiñedo et al., 2018). Incubation of wet mycelium in sterile distilled water (0.1 g / mL) was carried out with agitation on an orbital shaker (150 rpm). Then, the cell-free filtrate was collected by filtration through membrane filter with 0.45 μm pore size and 50 mL of a 5 mM AgNO_3_ solution were added to 50 ml of filtered solution. After incubation in dark the absorbance spectrum was measured in the range of 250–800 nm and the maximum peak was determined until no increase in the maximum absorption peak of silver nanoparticles was detected. The remaining cell-free filtrate was used as control.

### Purification of NPs

The synthesized NPs were centrifuged, washed and resuspended in sterile distilled water. Then, the absorption spectroscopy was performed. The absorbance band corresponding to the purified NPs was measured and their concentration was estimated as described by Paramelle et al. (2014).

### Characterization of NPs

#### Transmission Electron Microscopy (TEM)

An aliquot of NPs was placed onto a copper grid coated with a carbon film and dried for several hours at room temperature. TEM analysis was carried out in a TECNAI T20 electron microscope (FEI) working at 200 kV. Several micrographs using an automatic image analyzer (Image J Software) were analyzed to determine the average size of the NPs.

#### Confocal Raman Microscopy

For analysis by Confocal Raman Microscopy an aliquot of NPs was deposited on an aluminum support and dried at room temperature. An Alpha 300 RA WITec Raman Microscope using a 532 nm excitation laser focused through a 100 X objective was used for measurements.

#### Dynamic Light Scattering (DLS) and ζ Potential

The hydrodynamic diameter by Dynamic Light Scattering (DLS) and the measurement of the ζ potential of the NPs were determined using a Zetasizer from Malvern Instruments. The samples were prepared in Milli-Q water at pH 6. For the determination of DLS, each sample was measured 10 times, combining 5 runs per measurement, at 25°C. In the case of potential ζ, each sample was measured 3 times, combining 10 runs per measurement, at 25°C. Malvern’s Zetasizer software was used for processing the results.

### Antimicrobial Activity

#### Determination of Minimum Inhibitory Concentration (MIC) and Minimum Bactericidal Concentration (MBC)

Both MIC and MBC were determined by standardized methods, according to the Clinical and Laboratory Standards Institute (CLSI, 2015). The initial solutions of the NPs were prepared in sterilized distilled water. MIC of the nanoparticles was determined by the broth microdilution method in a 96-well plate (300 μL capacity, sterilized, MicroWell, NUNC, Thermo-Fisher Scientific, Waltham, MA), against *Salmonella* Typhimurium strains (Table 1). The MIC was determined as the lowest concentration of NPs that inhibited the visible bacterial growth after 24 h of incubation. Then, the broths used for MIC determination were subcultured onto fresh agar plates. After incubation, the number of viable cells was estimated by determining the number of colony-forming units (cfu). Based on this, the MBC was determined as the concentration of antimicrobial agent that causes the death of 99.9% of the initial inoculum, as previously reported (Díaz-García et al., 2020).

#### Confocal Raman Microscopy

The comparison of the phenotypic profiles of bacterial cells before and after treatment with NPs were performed using Confocal Raman Microscopy, according to the methodology previously reported for antibiotics by Athamneh et al. (2014). The bacterial cell suspensions were deposited on an aluminum support, dried at room temperature, and then measured using a 532 nm laser focused through a 100 X objective. The data processing and statistical analysis were performed, using a script of the research group, by principal component analysis (PCA) (Estevez et al., 2019). The phenotypic profiles (Raman spectra) of the bacterial cells treated with the NPs were compared to those of the untreated cells (control).

#### Atomic Force Microscopy

Bacterial Cells without treatment (control) and treated with NPs were observed by Atomic Force Microscopy (AFM), based on Borowik et al. (2018). Samples were analyzed using a WITec Alpha 300-RA AFM Microscope (WITec GmbH, Ulm, Germany), in the AC (tapping) mode. AFM measurements were obtained by placing a droplet of the bacterial suspensions on a silicon wafer substrate, dried at room temperature. AFM sensors had a force constant of *k* = 42 N/m, nominal resonance frequency of 285 kHz, mean width 45 μm, length 160 μm and thickness of 4.6 μm.

## Results

### Biological Synthesis of NPs

**FIGURE 1:**
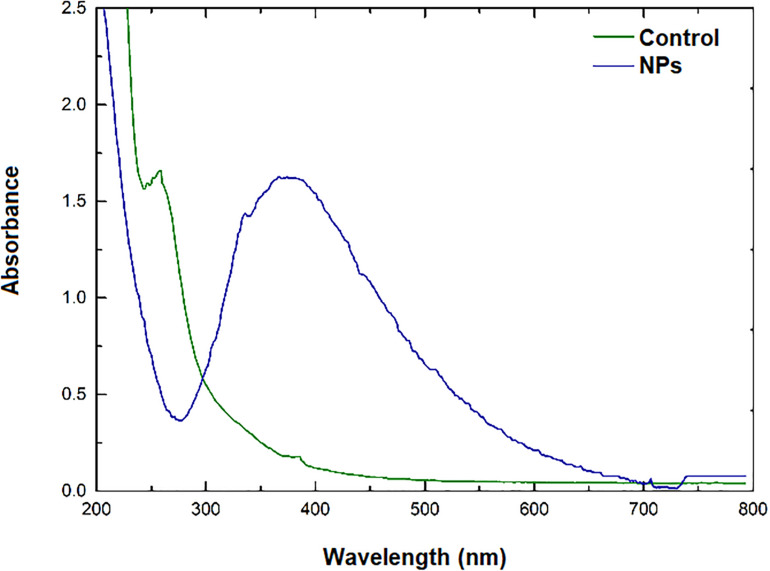
UV-Vis absorption spectrum after 24 h reaction.

After *Phanerochaete chrysosporium* biomass was harvested, incubated in water, and filtered, the cell-free filtrate was added to a silver nitrate solution and the mixture was incubated in dark. The synthesis of the silver NPs was monitored through changes in the absorbance spectra over time in the range of 250–800 nm, as well as color change in the reaction mixture. The appearance of an absorption band (maximum peak) at 400 nm corresponding to the surface plasmon resonance (SPR) and visible color change were indicative of the formation of NPs (Figure 1).

After a 24 h period the reaction mixture was centrifuged and the pellet containing NPs was washed with and resuspended in water. The UV-vis spectra after centrifugation showed an absorption band at 400 nm, corresponding to the SPR band of the purified NPs.

### Characterization of NPs

#### Transmission Electron Microscopy (TEM)

**FIGURE 2:**
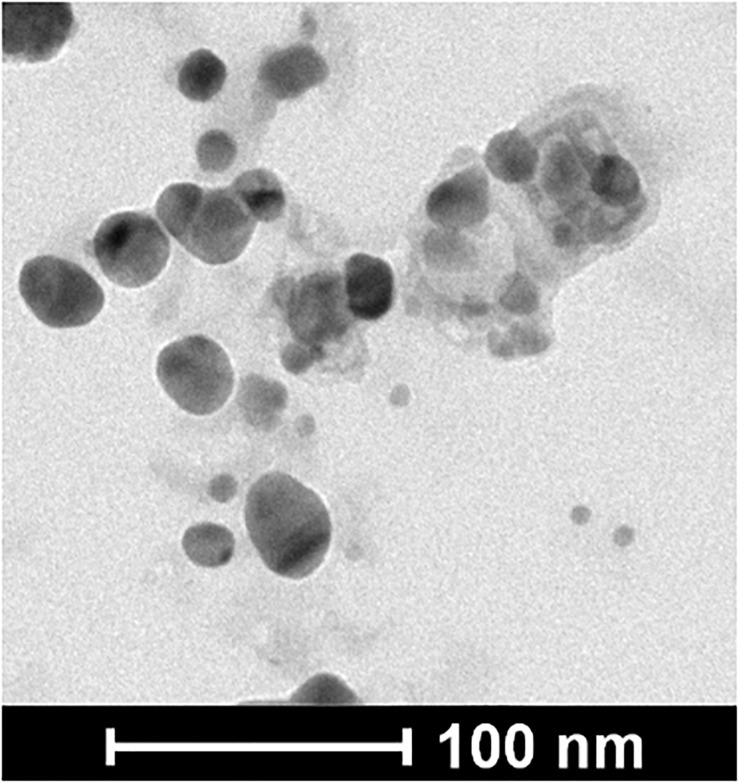
TEM image of the NPs.

The TEM characterization showed that the NPs were spherical and the image analysis using the Image J program resulted in an average size of NPs of 22 ± 6 nm (Figure 2).

#### Dynamic Light Scattering (DLS) and ζ Potential

Size characterization of NPs by DLS showed a single population with a hydrodynamic diameter of 47.64 ± 17.42, PDI 0.264, showing a slightly polydisperse distribution (<0.4), consistent with previously reported by Sanguiñedo et al. (2018).

**FIGURE 3:**
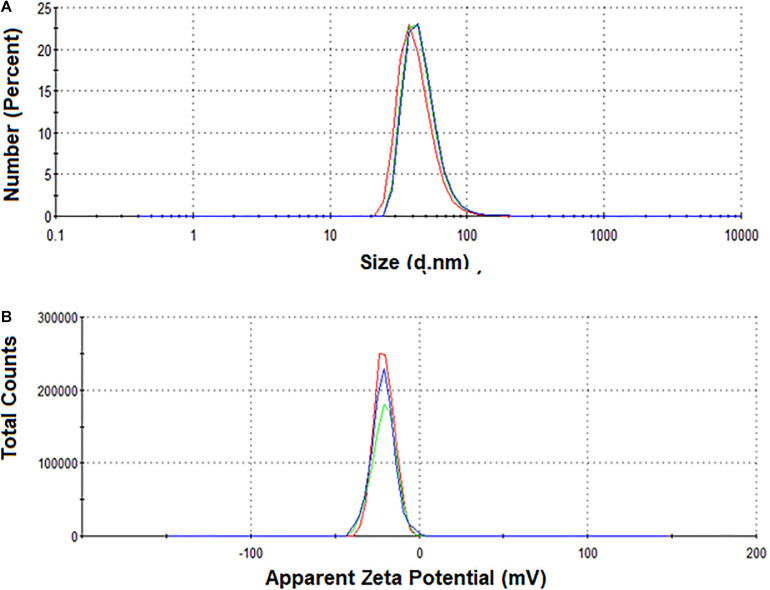
**(A)** DLS and **(B)** ζ-potential measurements of NPs in solution.

Measurements of ζ-potential showed that NPs had a high net negative surface charge, −20.7 ± 5.89 mV (Figure 3).

### Antimicrobial Activity

#### Determination of Minimum Inhibitory Concentration (MIC) and Minimum Bactericidal Concentration (MBC)

Table 2 shows the MIC values obtained for the NPs and the AgNO_3_ solution against the different strains of *Salmonella* Typhimurium. Although the strains were sensitive to the AgNO_3_ solution (39–78 μM), the MIC values obtained for the NPs were much lower (8–16 picomolar, pM). This difference is even greater when comparing the MBC values of the nanoparticles and the silver nitrate solution. The MBC values of the AgNO_3_ solution were greater than or equal to 830 μM, compared to the MBC values (16 pM) of the NPs.

**TABLE 2:** Minimum inhibitory and minimum bactericidal concentration of NPs and AgNO_3_ solution against *Salmonella* Typhimurium strains.

**STRAIN**	**MIC NPs**	**MBC NPs**	**MIC AgNO_3_**	**MBC AgNO_3_**
16-025M4	7.8 pM (0.3 ppm)	>15.6 pM	39 μM	>830 μM
16-045M5	7.8 pM (0.3 ppm)	15.6 pM (0.6 ppm)	39 μM	830 μM
16-058M4	15.6 pM (0.6 ppm)	15.6 pM (0.6 ppm)	39 μM	830 μM
16-078M4	7.8 pM (0.3 ppm)	>15.6 pM	39 μM	830 μM
17-032M21A	15.6 pM (0.6 ppm)	>15.6 pM	78 μM	>1,250 μM
17081M3A	15.6 pM (0.6 ppm)	>15.6 pM	78 μM	1,250 μM
17-197M1	15.6 pM (0.6 ppm)	15.6 pM (0.6 ppm)	39 μM	1,250 μM
17-210M7B	15.6 pM (0.6 ppm)	>15.6 pM	39μM	>830 μM
19-027M2A	15.6 pM (0.6 ppm)	>15.6 pM	78 μM	1,250 μM
16-073M11	15.6 pM (0.6 ppm)	>15.6 pM	78 μM	>1,250 μM

#### NPs-Bacterial Interactions

**FIGURE 4:**
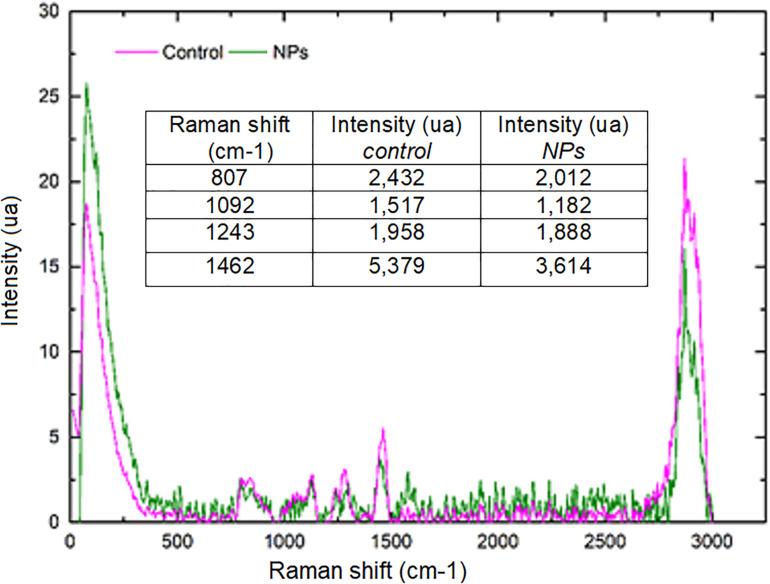
Comparison of average Raman spectra corresponding to *Salmonella* Typhimurium 17–197 M1 cells without treatment (pink) and treated with NPs (green). The intensity (in arbitrary units, AU) was normalized with respect to the signal intensity of the C-H band (2,900 cm^–1^ region). The decrease in the bands corresponding to RNA, carbohydrates and lipids after NPs treatment is shown.

**FIGURE 5:**
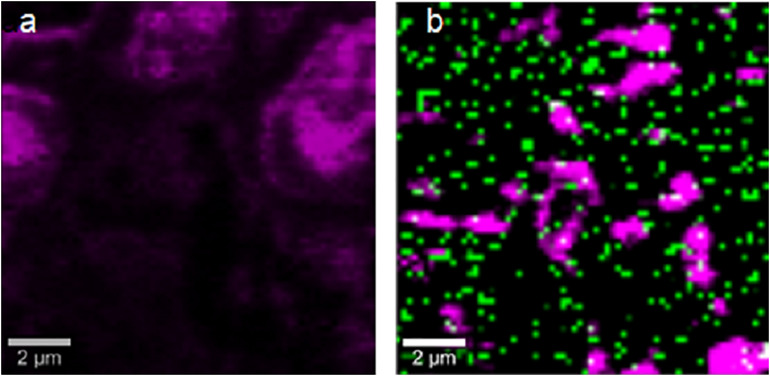
Confocal Raman images obtained by selecting the stretching C-H bands from the bacterial cells (pink) and the stretching Ag-N bands from the NPs (green). **(a)** Untreated cells. **(b)** NPs treated cells.

**FIGURE 6:**
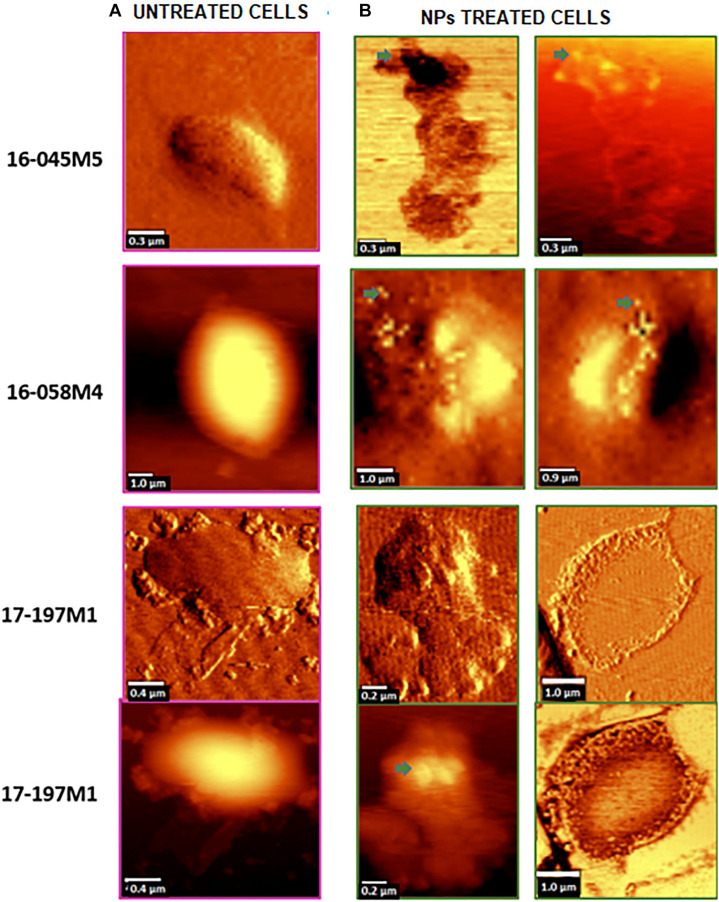
Topographic and phase contrast AFM images of changes in the cell morphology after exposure of *Salmonella* Typhimurium to NPs (indicated by arrows). **(A)** Untreated cells (control). **(B)** NPs treated cells.

The strains with the highest antimicrobial activity (the lowest MIC and MBC) were used for the Confocal Raman Microscopy studies (strains 16-045M5, 16-058M4, and 17-197M1). Raman band profiles previously reported for *Salmonella* spp. were compared to those obtained for the three strains. The presence of 11 coincident bands was observed. These bands were assigned to the corresponding biological molecules, as previously reported by other authors (Assaf et al., 2014; Witkowska et al., 2017; Table 3). Phenotypic changes in *Salmonella* Typhimurium cells treated with NPs were analyzed. Differences in Raman spectra due to phenotypic changes of cells treated with NPs versus untreated cells were determined (Figure 4), mainly based in the change of the intensity of the Raman bands. The data processing and statistical analysis were performed by principal component analysis (PCA). In all cases, it was observed that the interaction of NPs with *Salmonella* Typhimurium cells generates changes in the bands (particularly in their intensities), which were previously assigned to proteins, lipids, carbohydrates and nucleic acids. In addition, in order to offer a complementary study based on Raman, hyperspectral images were obtained with the purpose of visualizing the main characteristics of the interaction between the bacteria and NPs (Figure 5). For that reason we selected one characteristic Raman band from the bacterial cells and one from the NPs, these correspond to the C-H stretching and Ag-N stretching modes, respectively. Then we obtained an image in which the intensity of the C-H and Ag-N bands are integrated (total number of counts), then showing the more representative regions (topography) of the bacterial cells (in pink, Figures 5a,b) and NPs (in green, Figure 5b). Thus, we are providing a chemical image based in the chemical signature of both bacterial cells and NPs. Additionally, the Atomic Force Microscopy images indicate changes to cell morphology and loss of integrity of the cell envelope and of cytoplasmic content after exposure to the NPs (indicated by arrows) (Figure 6).

**TABLE 3:** Raman band assignment of bacterial cells (control).

**RAMAN SHIFT (cm^–1^)**			**ASSIGNMENT**
**16-045M5**	**16-058M4**	**17-197M1**	
557	556	545	Carbohydrates
709	745	713	DNA, RNA
757	761	796	DNA, RNA
800	810	807	RNA
935	970	934	Lipids
1,098	1,079	1,092	Carbohydrates
1,234	1,248	1,243	RNA
1,350	1,353	1,354	Proteins
1,444	1,448	1,462	Lipids
1,554	1,512	1,510	Proteins
1,594	1,575	1,579	DNA, RNA

*The band assignment was made based on previously published spectral data for strains of Salmonella spp. (Assaf et al., 2014; Witkowska et al., 2017).*

## Discussion

The silver nanoparticles synthesized with *Phanerochaete chrysosporium* extracts were stable after their purification by centrifugation, as previously reported by our group (Sanguiñedo et al., 2018).

The size of the NPs was determined by Transmission Electron Microscopy (TEM) and Dynamic Light Scattering (DLS). The average size of the NPs by Transmission Electron Microscopy (TEM) was 22 ± 6 nm. In addition, the characterization by DLS showed a larger average size (47.64 ± 17.42 nm), which is expected since the DLS measures the hydrodynamic diameter of the particles. Dispersity Parameter (PDI) showed a moderate polydisperse distribution (<0.4) from DLS results, consistent with that previously reported (Sanguiñedo et al., 2018). ζ-potential measurements demonstrate the colloidal stability of the NPs since they had a high net negative surface charge. The electrostatic repulsive forces between surface charges of NPs play an important role in the stability. Nanoparticle suspensions that show net potential close to zero, thus close to neutrality, probably add, losing their properties as nanomaterials due to the possibility of precipitation or aggregation (Joseph and Singhvi, 2019). Furthermore, the NPs used here were stable for at least 6 months at 4°C. The surface functional groups, capping of the silver nanoparticles, were characterized by Confocal Raman Microscopy. As previously reported, the presence of the band positioned at 230 cm^–1^ and Raman bands positioned at the fingerprint region can be attributed to the Ag-N stretching mode and to the presence of amino acids, respectively, suggesting that proteins from the fungal extract are part of the capping (Estevez et al., 2020).

The application of biogenic NPs as antimicrobial agents against resistant and multi-resistant *Salmonella* Typhimurium strains previously isolated from calves with diarrhea, septicaemia, and mortality was evaluated. The *in vitro* antimicrobial activity assays showed interesting results. In the MIC determination test, in addition to the strains under study, a reference strain *Salmonella* Typhimurium ATCC 14028 was used. However, no growth inhibition of this strain was observed at the concentrations evaluated. Therefore, we did not include it in subsequent trials where our objective was to evaluate the strains where growth inhibition was observed at these low concentrations. Perhaps this strain was sensitive to higher concentrations or it could have some particular resistance mechanism, as reported in other studies carried out with *Salmonella* spp. (Losasso et al., 2014). Results of antimicrobial activity against resistant *Salmonella* Typhimurium bacteria showed MIC and MBC values lower than silver nitrate solution. Although the resistance of *Salmonella* Typhimurium to silver ions has been reported (Randall et al., 2015), the properties of the biogenic silver NPs evaluated here would provide new antimicrobial advantages. Antibacterial activity of silver ions has been recognized since ancient times and although the antimicrobial action mechanism of silver ions is known, in the case of nanoparticles these mechanisms depend on the characteristics of the nanoparticles (Hamad et al., 2020). The intrinsic properties of metal nanostructures depend on their size, shape, composition, crystallite which is strongly correlated to their synthesis conditions (Hamad et al., 2020). In addition to the properties of the nanometric scale, that can confer higher penetration and larger surface area, capping agent of biogenic nanoparticles used here could provide stability and new antimicrobial properties. Although all strains are resistant to at least one antibiotic, all were sensitive to biogenic silver NPs. These results are particularly promising for the application of nanoparticles as an alternative treatment for fluoroquinolone resistant strains. Despite the use of fluoroquinolones was recommended for *Salmonella* Typhimurium in a recent work carried out with more than 11,000 strains (Wang et al., 2019), it would not be effective for the treatment of some strains isolated in Uruguay (Casaux et al., 2019). Furthermore, the nanoparticles showed high bactericidal activity (MBC 16 pM) against three multi-resistant *Salmonella* Typhimurium strains. Then, these strains were used for Confocal Raman Microscopy and Atomic Force Microscopy studies.

Raman spectroscopy is a powerful vibrational spectroscopic tool that can provide information on complex systems such as microbial cultures, through the determination of molecular fingerprints on various chemical and biochemical components in that systems. Compared to other methods that do not require culture such as the use of fluorescent or magnetic probes, Confocal Raman Microscopy has the unique potential of being a technique for phenotypic identification that does not require any particular treatment of cells (Ho et al., 2019). In this work, the previously reported Raman band profiles for *Salmonella* spp. were compared to those obtained for the three multi-resistant strains, showing 11 coincident bands, which were assigned to the corresponding biological molecules.

Moreover, Confocal Raman images of treated cells showed the colocalization of the NPs and the bacterial cell, by combination of stretching Ag-N bands assigned to the NPs and C-H stretching bands assigned to the bacterial cells. This imaging technique combined with principal component analysis showed that there are significant changes in the phenotypic profile of the NPs treated cells compared to the untreated cells (control). These results suggest that NPs cause changes in cellular composition, in carbohydrates, lipids, proteins and nucleic acids. These changes in the profile of the Raman spectra in *Salmonella* Typhimurium strains could be associated with damage at the protein, lipid and nucleic acid level produced by the generation of reactive oxygen species (ROS) and intermediate nitrogen species (RNI), since they are extremely toxic, and may be responsible for the death of bacterial cells (Durán et al., 2016). These results were complemented by Atomic Force Microscopy. AFM images showed changes in the cell morphology, loss of integrity of the cell envelope and of cytoplasmic content. Similar results using electron microscopy (TEM and ESEM) were recently reported for Gram negative bacterial cells (*E. coli*), after exposure to biogenic silver nanoparticles (Estevez et al., 2020). The properties of silver nanoparticles are strongly influenced by their size, distribution, morphological shape, and surface properties which can be modified by diverse synthetic methods, reducing agents and stabilizers. In spite of several hypotheses available, the antibacterial mechanisms of silver nanoparticles so far have not been established clearly (Lee and Jun, 2019). Some proposed cytotoxic mechanisms can be: adhesion of silver nanoparticles onto the membrane surface of microbial cells, modifying the lipid bilayer or increasing the membrane permeability; intracellular penetration of silver nanoparticles; nanoparticles-induced cellular toxicity triggered by the generation of reactive oxygen species (ROS) and free radicals, damaging the intracellular micro-organelles (i.e., mitochondria, ribosomes, and vacuoles) and biomolecules including DNA, protein, and lipids; modulation of intracellular signal transduction pathways toward apoptosis (Lee and Jun, 2019). According to our results in this work we think there would be more than one of the previously proposed mechanisms involved since we observe several morphological and structural changes in bacteria and that nanoparticles not only inhibit microbial growth but also kill them. With these promising results it would be interesting in future studies to deepen these mechanisms of action.

In conclusion, the results obtained in this work show the promising application of biogenic silver nanoparticles as a potential alternative treatment to fight against multi-resistant bacteria, relevant to human and animal health.

## Data Availability Statement

The raw data supporting the conclusions of this article will be made available by the authors, without undue reservation.

## Author Contributions

ME: methodology. MC: performing most of the experiments, selecting resistant *Salmonella* Typhimurium strains, and performing bacterial cultures. SA: writing—original draft preparation, supervision, and project administration. RF, MF, and ME: writing—review and editing. RF: supervising most of the experiments, supervising the nanoparticles characterization and Raman Confocal, and Atomic Force Microscopy studies. MF: supervising the selection and culture of the *Salmonella* strains. SA and RF: funding acquisition. All authors have read and agreed to the published version of the manuscript.

## Conflict of Interest

The authors declare that the research was conducted in the absence of any commercial or financial relationships that could be construed as a potential conflict of interest.
